# Real-world data-enabled, 2-randomisation, adaptive trial to determine the clinical efficacy and safety of widely used enteral feeds in preterm babies: the COLLABORATE trial – protocol

**DOI:** 10.1136/bmjopen-2026-125163

**Published:** 2026-10-06

**Authors:** Victoria Cornelius, James P Boardman, David Quine, Sabita Uthaya, Shalini Ojha, Hilary S Wong, Annemarie Lodder, Lauren Ingledow, Peter Bradley, James M S Wason, John Norrie, Ramon Luengo-Fernandez, Andrew Morris, Alex Baker, Ricardo Ribas, Daphne Babalis, Neena Modi

**Affiliations:** 1 Imperial Clinical Trials Unit, Imperial College London, London, UK; 2 Centre for Reproductive Health, University of Edinburgh Centre for Reproductive Biology, Edinburgh, UK; 3 Department of Neonatology, NHS Lothian, Edinburgh, UK; 4 School of Public Health, Imperial College London, London, UK; 5 School of Medicine, University of Nottingham, Nottingham, UK; 6 Department of Paediatrics, University of Cambridge, Cambridge, UK; 7 Adult Preemie Advocacy Network, London, UK; 8 Bliss, London, UK; 9 Population Health Sciences Institute, Newcastle University Population Health Sciences Institute, Newcastle upon Tyne, UK; 10 Centre for Public Health, Queen’s University Belfast, Belfast, UK; 11 Health Economics Research Centre, University of Oxford, Oxford, UK; 12 Health Data Research UK, London, UK

**Keywords:** NEONATOLOGY, Intensive Care Units, Neonatal, Randomized Controlled Trial

## Abstract

**Introduction:**

Enteral feed type is widely believed to affect brain growth and development directly as well as influencing the risk of necrotising enterocolitis (NEC), a leading cause of death and neuro-impairment in extremely preterm babies. Own mother’s milk (OMM) enhances neurodevelopment and reduces NEC. However, most babies born below 29 weeks gestation require some supplementary feeds during their neonatal unit stay because their mothers are unable to express sufficient milk. The available options, pasteurised human donor milk (pHDM) and preterm formula (PTF), differ markedly in nutrient and non-nutrient composition and cost. Non-definitive data suggest superiority of pHDM over PTF in relation to NEC risk, but no benefits have been shown in important corroboratory outcomes: surgical NEC, death, blood stream infection and neurodevelopmental impairment.

Human milk macronutrient content is variable and often low, hence some clinicians practise routine protein-carbohydrate fortification. Others fear that cow-milk derived fortifiers may increase NEC risk or may provide excessively high protein intakes dangerous to neurodevelopment and metabolic health. These uncertainties compromise patient care and safety and are strong justification for randomised evaluation.

**Methods and analysis:**

COLLABORATE is an efficient UK-wide, real-world data-enabled, 2-randomisation, group-sequential adaptive trial embedded in routine clinical practice. The primary outcome is survival to 34 weeks postmenstrual age without NEC surgery. The aim is to resolve two longstanding nutritional uncertainties for babies born below 29 weeks gestation: whether pHDM or PTF is the optimal supplement should there be an insufficiency of OMM; and whether OMM and pHDM require routine protein-carbohydrate fortification. Based on the assumption that 50% of participants will take part in both randomisations, total recruitment is targeted at 3252 (5% between-arm primary outcome difference; α=0.05; minimum 87% power).

**Ethics and dissemination:**

COLLABORATE is funded by the UK National Institute for Health and Care Research, sponsored by Imperial College London and approved by the UK Health Research Authority (reference 25/LO/0697). Recruitment commenced in April 2026. Results will be shared through academic and lay publications, conferences, workshops, social media and strong personal networks.

**Trial registration number:**

ISRCTN10443084.

**Strengths and limitations of this study**

- This is the largest UK-wide comparative-effectiveness randomised controlled trial.
- The trial is operationally and statistically efficient with 2-randomisations, a group-sequential design, embedding in routine neonatal care and uses a source of quality-assured real-world data, the National Neonatal Research Database to minimise the burden of trial data acquisition.
- A digital version of the parent completed Parent Report of Children’s Abilities-Revised questionnaire is used to obtain 2-year language and cognitive outcomes.
- The open-label design may be vulnerable to reporting bias.

## Introduction

 Own mother’s milk (OMM) is an accepted standard of care for all newborn babies. However, the developmental ability to suckle effectively is not reached until 33–34 weeks postmenstrual age (PMA), hence mothers delivering preterm must express breast milk for many weeks which they widely report as stressful and difficult to sustain. Therefore, a majority of extremely preterm babies require supplementary feeds at some point during the neonatal unit in-patient stay. Overall, in the UK, around a third of extremely preterm infants requiring supplementary feeds receive pasteurised human donor milk (pHDM) and around two-thirds receive preterm formula (PTF).^1^ The optimal supplement is unknown.

This question is important as enteral feed types are widely considered to affect brain growth and development, either directly or indirectly by influencing the risk of necrotising enterocolitis (NEC). NEC is an acquired inflammation of the immature gastrointestinal tract and leading cause of death and life-long impairment in extremely preterm babies in high-income settings. There are around 3000 babies born <29 weeks’ gestation, and around 260 NEC deaths and/or surgeries each year in the UK, with up to three times more developing less severe disease that nonetheless disrupts feeding, prolongs hospital stay and increases antibiotic exposure, and costs of care.^2^

OMM reduces but does not wholly prevent NEC.^2^ OMM and pHDM are not equivalent as human milk is very variable in nutrient and non-nutrient content, composition is unique to each mother and pasteurisation substantially reduces or destroys biologically active non-nutritional components.^3^ PTF has consistent composition and pHDM nutrient concentration is often low as donors have usually delivered at full-term and have been lactating for several weeks. Some clinicians believe routine addition of macronutrient fortifier to OMM and pHDM is warranted. Others are concerned that this may expose some babies to excessively high protein intakes, dangerous to neurodevelopment, renal and metabolic health. In the UK, around half of extremely preterm babies receive fortified feeds.^1^

This study will address the longstanding unmet health need of the daily uncertainty faced by healthcare professionals and parents in the UK and around the world regarding the efficacy and safety of pHDM, PTF and fortifier in relation to NEC, survival and cognitive-language development. The study will also establish value for money. The costs of surgical treatment of NEC are high (~£16 500 per case), as are the societal costs associated with suboptimal neurodevelopment. There are also large cost differences between pHDM (£125–£200/L), PTF (£5/L) and fortifier (£5/L of human milk fortified). An embedded single-centre mechanistic study will provide insight into the effects of feed types on the developing preterm brain.

### Study justification

This research is needed because the uncertainties have led to a proliferation of non-evidenced based guidelines, variation in practice, anxiety for parents and risks to patient safety. Parents, patients (those living with the long-term consequences of neonatal conditions), clinicians and the James Lind Priority Setting Partnership have repeatedly ranked identification of optimal enteral feeding strategies a priority for well over a decade.^4–6^

The National Institute for Health and Care Excellence (NICE) guidance ‘Donor milk banks: service operation’ acknowledges the limited high-quality evidence for use of pHDM and includes the recommendation for research to evaluate efficacy in improving outcomes and identify babies who would benefit most.^7^ The 2023 British Association of Perinatal Medicine ‘Framework for use of Donor Milk’ also highlights the urgent need for this research.^8^ The American Academy of Pediatrics and European Society for Paediatric Gastroenterology, Hepatology and Nutrition only conditionally support use of pHDM as the supplementary feed of choice, acknowledging the benefits of OMM over pHDM, the variable nutrient content and uncertain non-nutrient benefits of pHDM, and the limited evidence-base for practice.

Further justification for the study that presents current evidence and safety considerations can be found in online supplemental methods section 1.

### Aims and objectives

COLLABORATE will address the following hypotheses, set out in PICO (P: population, I: intervention, C: comparator, PO primary outcome) format:

#### Randomised trial hypotheses

- In babies born <29 weeks’ gestation for whom there is need to supplement OMM because the volume available is insufficient (P), pHDM (I), when compared with PTF (C), improves survival rate to 34 weeks PMA without surgical NEC (PO).
- In babies born <29 weeks’ gestation (P), routine macronutrient fortification of human milk (OMM and pHDM) with cow milk fortifier (I), when compared with no routine fortification (C), improves survival rate to 34 weeks PMA without surgical NEC (PO).

#### Mechanistic hypotheses

- In babies born <29 weeks’ gestation for whom there is a need to supplement OMM because the volume available is insufficient (P), pHDM (I), when compared with PTF (C), differs in effect on the maturation of cerebral white matter tracts that subserve cognitive function (PO), assessed at full-term.
- In babies born <29 weeks’ gestation for whom there is a need to supplement OMM because the volume available is insufficient (P), the use of pHDM (I) compared with PTF (C) results in a different gut microbiota composition and/or diversity (PO).

#### National Health Service (NHS) value hypothesis

- In babies born <29 weeks’ gestation for whom there is a need to supplement OMM because the volume available is insufficient (P), the additional cost of pHDM (I), when compared with PTF (C), generates savings through improved survival to 34 weeks PMA without surgical NEC (PO).
- In babies born <29 weeks’ gestation (P) who receive routine (I) versus no routine (C) fortification of human milk feeds, the additional cost of fortifier generates savings through improved survival to age 2 years corrected for preterm birth without moderate-severe cognitive-language impairment (PO).

### Outcomes

The primary outcome is survival without surgical NEC to 34 weeks PMA. Secondary outcomes are shown in table 1. Further details of other study endpoints and schedule of assessments are shown in online supplemental tables 1 and 2.

**Table 1:** List of secondary outcomes

Assessed at neonatal unit discharge	Assessed at 2 years corrected for preterm birth
Survival	Survival without moderate-severe cognitive language
Surgical NEC	Parent Report of Children’s Abilities-Revised (PARCA-R)
Spontaneous intestinal perforation	
Bronchopulmonary dysplasia	
Age in days to achieve enteral intake of 150 mL/kg/day	
Treated retinopathy of prematurity	
Severe brain injury	
Diagnosis of milk-curd obstruction	
Length of neonatal unit stay	
Number of episodes of bacterial or fungal bloodstream infection	
Number of episodes of bacterial or fungal cerebrospinal fluid infection	
Number of episodes of bacterial or fungal urinary tract infection	
Number of days of antibiotic treatment	
Medical NEC	

NEC, necrotising enterocolitis.

## Methods

### Design

#### Main study

A UK-wide, real-world data-enabled, 2-randomisation, group-sequential, open-label, adaptive, comparative-effectiveness trial embedded in routine clinical care (figure 1) with an internal pilot (table 2). Co-enrolment with other trials is permitted. The principal data source is the National Neonatal Research Database (NNRD) that contains a quality-assured extract from electronic patient records. Data storage and analysis will be conducted at the Imperial Clinical Trials Unit. Interim efficacy analyses can be found in online supplemental table 3.

**Figure 1:**
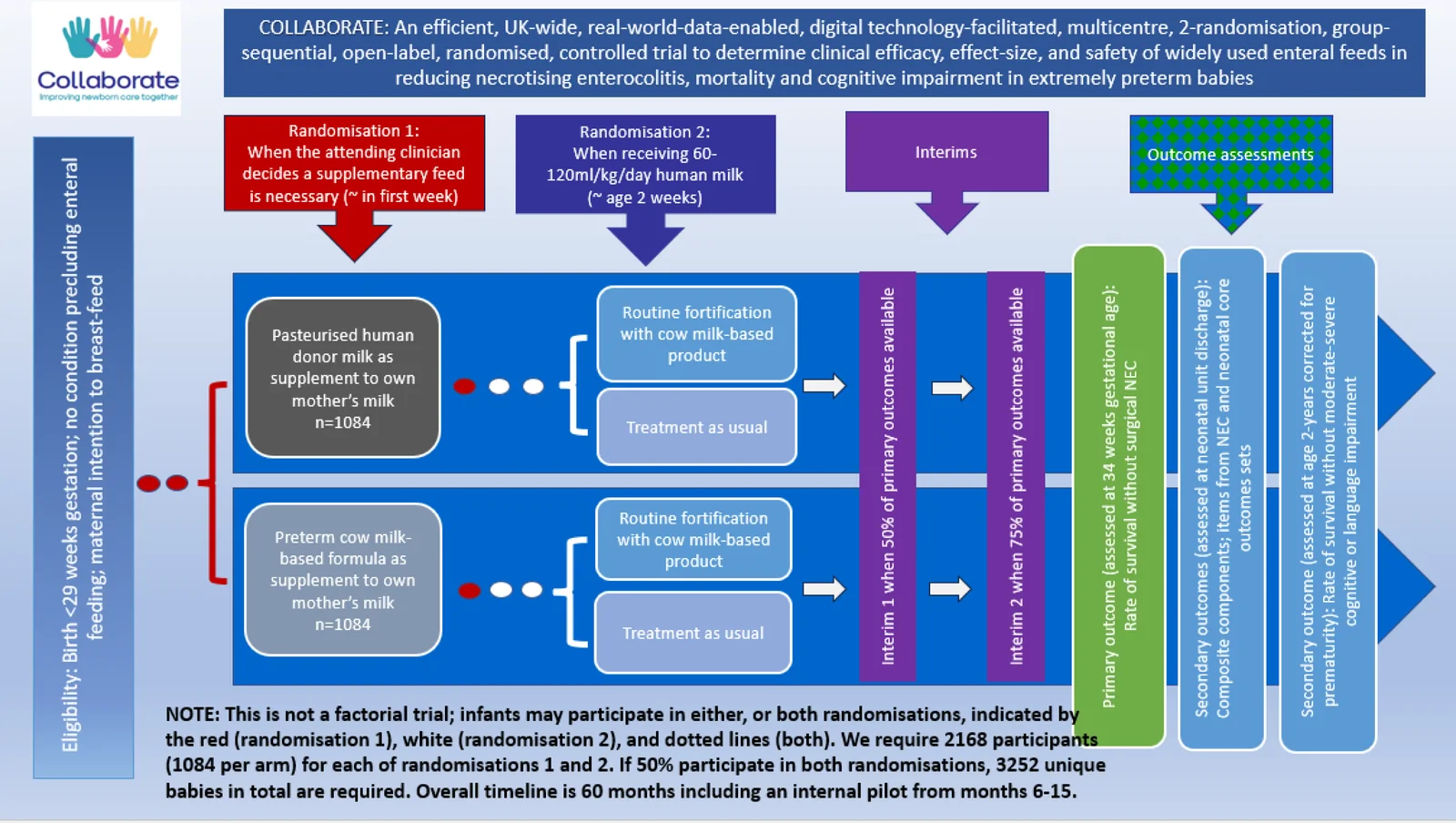
Study flow chart. NEC, necrotising enterocolitis.

**Table 2:** Internal pilot to assess recruitment rate, co-enrolment rate and withdrawal of consent rate

Progression criteria	Recruitment per comparison	Proportion taking part in both trials	Withdrawn consent for use of NNRD data
**RED: consider stopping**	<322 randomisations in each comparison	<30% (as trial will have <80% power)	>10%
**AMBER: explore approaches to achieve target**	322–644 randomisations in each comparison	30%–50%	>2% but < 10%
**GREEN: continue without changes**	>644 recruited to each comparison	>50%	≤2%

NNRD, National Neonatal Research Database.

#### Mechanistic study

An embedded mechanistic, single-centre, substudy that will use MRI to determine whether pHDM and PTF have different effects on cerebral white matter maturation, the hallmark feature of encephalopathy of prematurity, and hence impact the development of brain networks that underpin cognition. We will explore the potential differential effects of the interventions on cortical morphology and microstructure. This substudy will involve 120 infants participating in COLLABORATE at the Royal Infirmary of Edinburgh. Based on computational modelling and prior precedent from neonatal neuroprotection trials, a study of 60 infants in each treatment arm will enable detection of a 10% difference in fractional anisotropy with 80% power, two-sided 5% significance.^9^ See online supplemental methods section 3 for more information.

#### Cost-effectiveness study

COLLABORATE embeds a within-trial economic evaluation to establish value for money of pHDM versus PTF, due to the large cost difference between the two interventions (up to £200/L for pHDM versus £5/L for formula) and the high costs of NEC treatment.

### Setting

UK neonatal units.

### Population

#### Inclusion criteria

Babies born less than 29 weeks’ gestation with no condition precluding enteral feeding and maternal intention to express breast milk.

#### Exclusion criteria

Babies are excluded from randomisation 1 if they have already received pHDM or PTF and from randomisation 2 if they have already received fortifier.

### Consent

Members of the neonatal unit clinical team will seek verbal informed consent for the main study and written consent for the mechanistic substudy.

### Randomisation

Babies may participate in either or both randomisations. Randomisation 1 will occur when the attending clinician decides a supplemental feed is required because the volume of OMM is insufficient. Randomisation 2 will occur when the baby is receiving between 60 and 120 mL/kg/day of human milk feeds (OMM and/or pHDM).

Randomisation will be carried out using an online system managed by Imperial Clinical Trials Unit using minimisation (with 10% random allocation) to achieve balance in each treatment group over well-recognised prognostic factors (neonatal network (n=13); gestational age category (<26 weeks; ≥26 weeks); severe growth restriction (<10% centile; ≥10% centile based on standard criteria)). Multiple births will be randomised individually in keeping with our feasibility study,^10^ other feeding trials^11^ and parent focus group advice. Blinding is impracticable as each of the interventions in randomisation 1 are easily recognisable and because fortifier is added to feeds immediately before administration.

#### Nutritional supplements

We recommend that infants randomised to the ‘no routine fortification’ arm receive routine oral phosphate supplements to protect against metabolic bone disease. Other supplements including vitamins will be provided in accordance with the policies of the participating neonatal unit.

#### Discretionary fortification

Experience from the COLLABORATE feasibility study, PREMFOOD, indicates that the need for rescue fortification is likely to be minimal.^12^ However, clinicians may consider discretionary use of fortifier in babies participating in COLLABORATE randomisation 2 if after regaining birth weight, and receiving a human milk intake of 180 mL/kg/day or more, a baby shows a persistent downward crossing of anthropometric centiles over a 10-day period on the UK Neonatal and Infant Close Monitoring growth chart and the trend in blood urea is falling, with a value below 1.5 mmol/L indicating no protein excess and likely protein deficiency and chronic sodium depletion has been excluded (chronic sodium depletion is manifest by a slowing of weight gain despite a human milk intake of 180 mL/kg/day or more, noting that the serum sodium concentration in early deficiency will be normal) and there is no severe intercurrent illness. Discretionary fortification may also be considered if a baby is unable to tolerate a human milk volume of 180 mL/kg/day (eg, treatment resistant gastro-oesophageal reflux; high output stoma) and the conditions above apply. Clinicians considering discretionary fortification are advised to discuss this with the clinical chief investigator or delegate. Discretionary fortification will be documented in the electronic Case Record Form (eCRF) and factored into analyses.

#### Follow-up

Babies will be followed up until discharge and again at 2 years for cognitive and language outcomes measured using the parent-completed Parent Report of Children’s Abilities-Revised (PARCA-R) in accordance with NICE recommendation.^13^ A digital version of the PARCA-R will be made available to clinical teams should they wish to use this.^14^

### Discontinuation of study interventions and study withdrawal

Participant parents/guardians may discontinue study interventions by request. The attending clinician may discontinue participation if it is considered, following discussion with the Chief Investigator or authorised delegate**,** that the participant’s health would be compromised by continuation. The reason for discontinuation will be recorded in the eCRF and medical records. Participants will continue to be followed up even if the intervention has been discontinued.

### Sample size and statistical analysis

This is a group-sequential trial with two interims and one final analysis. The maximum sample size is 3252 (n=2168 in each randomisation; n=1084 per arm in each randomisation) assuming no early stopping, estimated on the composite primary efficacy outcome (survival to 34 weeks gestational age without surgical NEC (Y/N)).

For the randomisation 1 (pHDM vs Formula supplement), an observational analysis of NNRD data estimated the difference in the proportion surviving to 34 weeks’ PMA for babies born <29 weeks’ gestation to vary between 0.18 and 0.52 depending on week of gestational age (favouring supplementation with PTF).^15^ For the sample size calculation, we targeted a conservative Minimum Clinically Important Difference (MCID) in this proportion of 0.05 based on a baseline of 0.82 which we accurately estimated from the NNRD for this population of babies in the UK.

For randomisation 2 (fortifier vs no routine fortifier), an unpublished observational analysis from NNRD data estimated the difference in proportion data surviving to 34 weeks’ PMA for babies born <29 weeks gestation to vary between 0.06 and 0.75 depending on week of gestational age (favouring fortifier). Taking a conservative approach and in line with the first comparison, we have chosen to target the same MCID of 0.05.

We calculated power based on a fixed sample size of 3252 unique babies using rpact^16^ where interim timings were chosen to minimise the expected sample size. We anticipate at least 50% of babies will be co-enrolled into both randomisations. If co-enrolment is higher, power will increase. We have used an O’Brien and Fleming design with two-sided overall significance level of 5% to account for two interims at 50% and 75% and a normal approximation assumption for rates. For both randomisations we have factored in 2% missing outcome data due to carers withdrawing consent from routine data collection in NNRD. Based on all the above assumptions, n=3252 (n=2168 in each randomisation and n=1084 per arm) will achieve a minimum of 87% power for each randomisation. The expected sample size across for each randomisation is n=2439.

A treatment policy estimand will be targeted to estimate the effect of each intervention as delivered in practice regardless of any events that occur post-randomisation. The main intervention effect for each randomisation will be modelled separately using a binomial Generalised Estimating Equation (GEE) model with identity link and a log link function to estimate adjusted risk difference and adjusted risk ratio with robust variance. This will enable us to account for within network correlations. Intervention effect for the first and second randomisation will be modelled separately. Both models will be adjusted for minimisation variables.

The analysis model for randomisation 1 will include whether an infant received fortification (Y/N) and whether or not they were randomised to this (randomisation 2; Y/N). Though randomisation 2 will occur after randomisation 1, they are close in time and receiving fortification may be more likely to be a predictor of the primary outcome than a result of taking part in randomisation 1. Therefore, randomisation 2 and receipt of fortification will be treated as baseline covariates.

The analysis model for randomisation 2 will include adjustment for taking part in randomisation 1 (Y/N) and type of supplementary feed received (pHDM or formula). A supplementary analysis for randomisation 2 will be performed to determine the effect of rescue fortification in the standard of care arm (no routine fortification) will be undertaken using principal stratum and complier average causal effect approaches (no rescue fortification is defined as a complier in the standard of care arm). In the unlikely case that missing data are >2%, a sensitivity analysis will be performed using multiple imputation.

Binary secondary and key safety outcomes (death, surgical NEC, medical NEC, infection) will be analysed using the same principled approach as the primary outcome. The age 2-year PARCA-R will also be evaluated as a continuous outcome and the between-arm mean difference estimated using the GEE model with normal distribution and identity link function adjusting for minimisation variables. No adjustment will be made for multiple testing across secondary outcomes; consequently, these analyses will be regarded as supportive and hypothesis-generating rather than confirmatory.

#### Subgroup analyses

We will perform subgroup analyses for the primary outcome by gestational age category (≤26 weeks; >26 weeks) and growth centile (<10% centile; ≥10% centile). The subgroups will be examined through use of an interaction term between the subgroup and the intervention arm. Results will be judged at an alpha of 0.20, presented as forest plots, and will be hypothesis generating.

#### Interim assessment of efficacy

We will conduct two pre-planned formal interim efficacy analyses when 50% and 75% of participants reach 34 weeks gestational age (ie, primary outcome is available) (online supplemental table 3). This analysis will be undertaken by an independent ICTU statistician. Recruitment will continue during interim analyses. The analysis will employ two-sided O’Brien-Fleming efficacy boundaries. Unadjusted between-arm differences in proportions will be calculated using the primary analysis models described for the final analysis. An identity link function will be used to obtain adjusted risk differences and corresponding Z-scores. Superiority will be assessed at each stage based on the critical value thresholds (Z-scores) and numbers of participants.

The analysis for the internal pilot, exploratory analysis and safety analysis is specified in online supplemental methods section 4.

#### Mechanistic substudy

We will use Tract-based Spatial Statistics optimised for neonates to compare voxel-wise statistics across the white matter skeleton between treatment groups using a general linear model adjusting for gestational age at birth, age at image acquisition and any important covariates that remain imbalanced after randomisation.^17 18^

#### Cost effectiveness study analyses

NHS secondary care utilisation from randomisation until neonatal unit discharge will be obtained from the NNRD, as used in other successful economic evaluations.^19 20^ Further details of the cost analysis can be found in online supplemental methods section 5.

#### Analysis commencement

The study analysis is due to start Spring 2028.

### Parent, patient, public involvement and engagement (PPPIE)

The study PPPIE lead will ensure continuing involvement supported by co-investigators Bliss (the national preterm and sick baby charity), APAN (Adult Preemie Advocacy Network), collaborating organisation NEC UK (national charity) and community partners, people with experience of neonatal care that have strong links with their communities. We have recruited a PPPIE advisory group of 8 mothers of babies born preterm and/or with experience of NEC to offer advice throughout the study. All trial committees will include PPPIE representatives.

PPPIE has played an integral part in the development of this proposal. Our work so far has shown that parents and former neonatal patients have a good understanding of the uncertainties we aim to resolve. Their contributions helped develop the study in several ways.^21–23^

Mothers relayed the pressure they experienced to express breast milk. Focus groups identified the importance of using language that does not add to guilt when unable to provide enough. Some parents expressed distaste at the thought of donor milk; others worried about formula.

Focus groups also elicited the novel finding that many parents, especially those with experience of NEC, felt the opportunity to participate in a randomised trial to resolve feeding uncertainties would relieve anxieties brought about by not knowing which care practice was best. Parents expressed strong altruism in wanting to benefit other babies. We will convey these insights to recruiting neonatal teams to ensure that discussions about expressing breast milk do not increase parent distress and that they are aware that rather than shying away from participation, parents want their babies and other infants to benefit through research.

Parents told us information leaflets should not be ‘dumbed down’ versions of scientific information but written honestly, with the full diversity of parents in mind. They helped us co-create a Parent Information Leaflet. Parents also suggested we provide short video clips voiced by parents that explain the study (https://www.imperial.ac.uk/neonatal-data-analysis-unit/collaborate/). The COLLABORATE logo was also co-developed in collaboration with a parent (figure 2).

**Figure 2:**
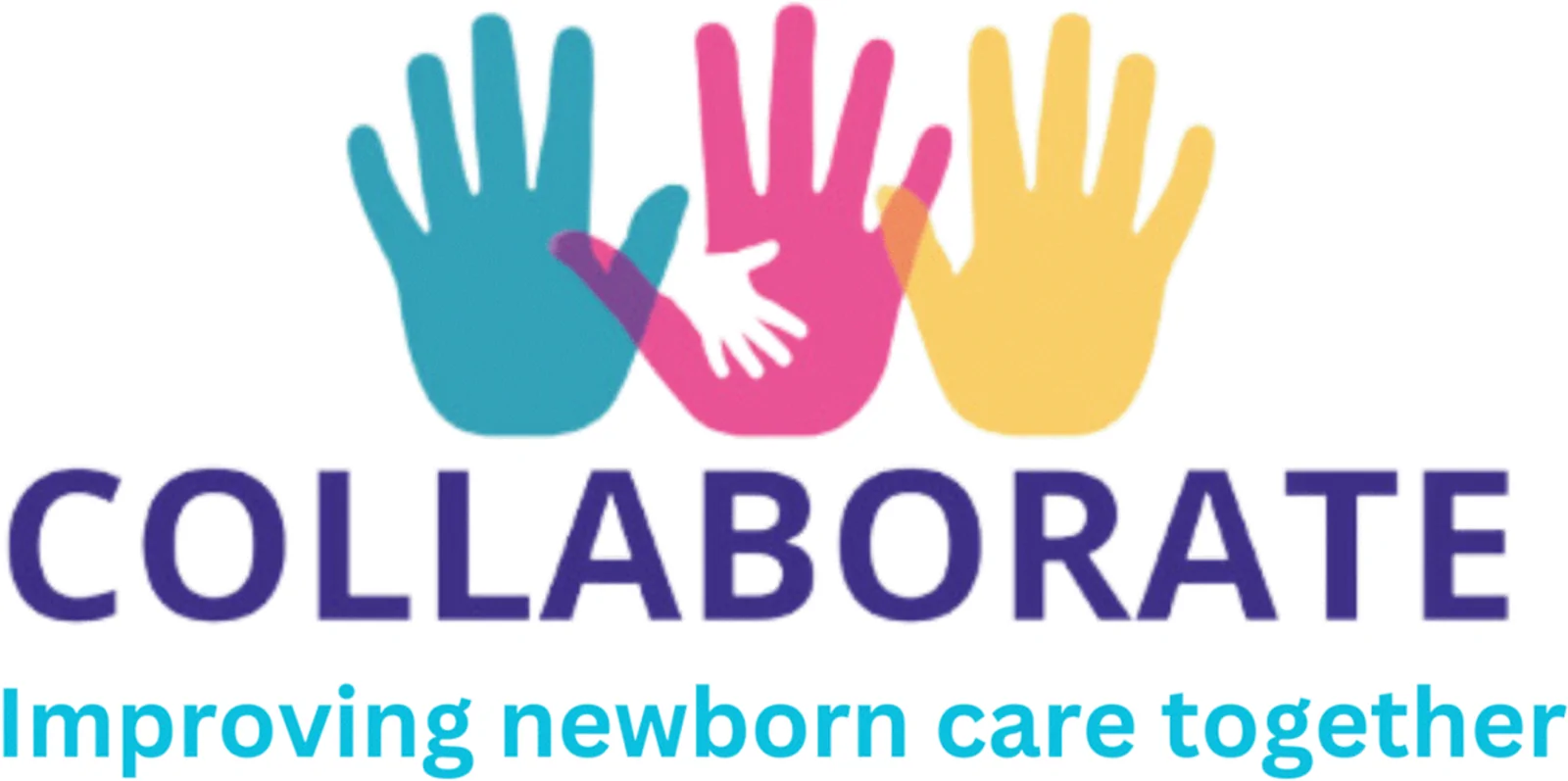
COLLABORATE logo.

### Trial committees

These comprise an independent Data Monitoring Committee and Trial Steering Committee with independent and non-independent members, constituted in accordance with standard NIHR guidelines. In addition, a Parent Advisory Group and a Scientific Advisory Group have been assembled to provide advice in relation to public-parent involvement, engagement and participation, and embedded substudies, respectively.

### Trial material and data access

The trial protocol and amendments and all other trial materials including DMC and TSC charter are either accessible on the COLLABORATE website or by request to the Imperial Clinical Trials Unit. Requests to access trial data may be made to the Chief Investigators through the Imperial Clinical Trials Unit following completion of the trial and publication of outcomes.

### Ethics and dissemination

COLLABORATE is approved by the UK Health Research Authority (reference 25/LO/0697). Recruitment commenced in April 2026. Safety is monitored throughout the study at regular intervals by an Independent Data Monitoring Committee. In addition to traditional peer-reviewed publications, we have planned activities to disseminate outputs through multiple routes to parents, NHS clinical teams, trainees, managers, commissioners, policymakers and NICE, as well as neonatal practitioners globally. We are in regular communication with every neonatal unit in the UK as they all contribute to the NNRD, providing us with additional means of providing information directly. We are especially committed to communicating with ‘hard-to-reach’ families who are over-represented in admissions to neonatal units.

## Supplementary material

10.1136/bmjopen-2026-125163 online supplemental file 1

## References

[1] Greenbury SF, Ougham K, Wu J (2021). Identification of variation in nutritional practice in neonatal units in England and association with clinical outcomes using agnostic machine learning. Sci Rep.

[2] Battersby C, Longford N, Mandalia S (2017). Incidence and enteral feed antecedents of severe neonatal necrotising enterocolitis across neonatal networks in England, 2012-13: a whole-population surveillance study. Lancet Gastroenterol Hepatol.

[3] Colaizy TT (2021). Effects of milk banking procedures on nutritional and bioactive components of donor human milk. Semin Perinatol.

[4] Evans K, Battersby C, Boardman JP (2023). National priority setting partnership using a Delphi consensus process to develop neonatal research questions suitable for practice-changing randomised trials in the UK. Arch Dis Child Fetal Neonatal Ed.

[5] Duley L, Uhm S, Oliver S (2014). Top 15 UK research priorities for preterm birth. Lancet.

[6] Oliver S, Uhm S, Duley L (2019). Top research priorities for preterm birth: results of a prioritisation partnership between people affected by preterm birth and healthcare professionals. BMC Pregnancy Childbirth.

[7] National Institute for Health and Care Excellence (2010). Donor milk banks: service operation. Clinical guideline [CG93]. Checked in 2018; no new evidence identified and no changes made.

[8] (2023). The use of donor human milk in neonates. https://www.bapm.org/resources/the-use-of-donor-human-milk-in-neonates.

[9] Ball G, Boardman JP, Arichi T (2013). Testing the sensitivity of Tract-Based Spatial Statistics to simulated treatment effects in preterm neonates. PLoS One.

[10] Brown JV, Lin L, Embleton ND (2020). Multi-nutrient fortification of human milk for preterm infants. Cochrane Database Syst Rev.

[11] Uthaya S, Jeffries S, Andrzejewska I (2022). Randomised controlled trial of human derived breast milk fortifier versus bovine milk fortifier on body composition in very preterm babies. Early Hum Dev.

[12] Mills L, Chappell KE, Emsley R (2024). Preterm Formula, Fortified or Unfortified Human Milk for Very Preterm Infants, the PREMFOOD Study: A Parallel Randomised Feasibility Trial. Neonatology.

[13] (2017). Developmental follow-up of children and young people born preterm.

[14] Modi N, Ribas R, Johnson S (2023). Pilot feasibility study of a digital technology approach to the systematic electronic capture of parent-reported data on cognitive and language development in children aged 2 years. *BMJ Health Care Inform*.

[15] Chehrazi M, Lanoue J, Ougham K (2023). Outcomes in very preterm infants receiving an exclusive human milk diet, or their own mother’s milk supplemented with preterm formula. Early Hum Dev.

[16] Rpact: confirmatory adaptive clinical trial design and analysis [program]: CRAN (The Comprehensive R Archive Network)..

[17] Smith SM, Jenkinson M, Johansen-Berg H (2006). Tract-based spatial statistics: voxelwise analysis of multi-subject diffusion data. Neuroimage.

[18] Ball G, Counsell SJ, Anjari M (2010). An optimised tract-based spatial statistics protocol for neonates: applications to prematurity and chronic lung disease. Neuroimage.

[19] Pillay T, Modi N, Rivero-Arias O (2019). Optimising neonatal service provision for preterm babies born between 27 and 31 weeks gestation in England (OPTI-PREM), using national data, qualitative research and economic analysis: a study protocol. BMJ Open.

[20] Yang M, Campbell H, Pillay T (2023). Neonatal health care costs of very preterm babies in England: a retrospective analysis of a national birth cohort. *BMJ Paediatr Open*.

[21] Moss B, Lammons W, Johnson S (2022). More than words: Parent, Patient and Public Involvement perspectives on language used by clinical researchers in neonatal care. Early Hum Dev.

[22] Lammons W, Moss B, Battersby C (2021). Incorporating parent, former patient and clinician perspectives in the design of a national UK double-cluster, randomised controlled trial addressing uncertainties in preterm nutrition. *BMJ Paediatr Open*.

[23] Lammons WB, Moss B, Bignell C (2023). Involving multiple stakeholders in assessing and reviewing a novel data visualisation tool for a national neonatal data asset. *BMJ Health Care Inform*.

