# Supplementary material for: Real-world data-enabled, 2-randomisation, adaptive trial to determine the clinical efficacy and safety of widely used enteral feeds in preterm babies: the COLLABORATE trial – protocol
Source: BMJ Open. 2026 Oct 6;16(10):e125163. doi: 10.1136/bmjopen-2026-125163 (PMC13646953; doi:10.1136/bmjopen-2026-125163)
Supplement: Supplementary file 1 [file bmjopen-2026-125163.reviewer_comments.pdf]

PEER REVIEW HISTORY

BMJ Open publishes all reviews undertaken for accepted manuscripts. Reviewers are asked to complete a checklist review form and are provided with free text boxes to elaborate on their assessment. These free text comments are reproduced below.

ARTICLE DETAILS

Title (Provisional)

Protocol for a real-world-data enabled, 2-randomisation, adaptive trial to determine the clinical efficacy and safety of widely used enteral feeds in preterm babies: The COLLABORATE trial

Authors

Cornelius, Victoria; Boardman, James P; Quine, David; Uthaya, Sabita; Ojha, Shalini; Wong, Hilary S; Lodder, Annemarie; Ingledow, Lauren; Bradley, Peter; Wason, James M. S.; Norrie, John; Luengo-Fernandez, Ramon; Morris, Andrew; Baker, Alex; Ribas, Ricardo; Babalis, Daphne; Modi, Neena

---

VERSION 1 - REVIEW

Reviewer 1

Name Lei, Xiaoping

Affiliation Affiliated Hospital of Southwest Medical University, Department of Neonatology

Date 24-Aug-2026

COI None

---

1. Individual infants may be exposed to two randomised interventions simultaneously, raising the possibility of an interaction between pHDM and human milk fortification status.

- The protocol only plans post-hoc subgroup interaction tests with an alpha threshold of 0.20 intended solely for hypothesis-generation. No formal power calculation for detecting interaction effects was pre-specified. Potential synergistic effects between the two interventions may partly distort estimates of main effects, which should have been anticipated a priori.

- Multiple interim analyses, two separate randomisation procedures and numerous secondary outcomes increase the risk of type-I error inflation. Only interim efficacy analyses employ the O'Brien-Fleming stopping boundaries; no multiplicity adjustment is mentioned for other secondary or exploratory analyses.

2. This study uses event-driven conditional randomisation whereby participants are dynamically screened according to their clinical course. Consequently, trial findings can only

be generalised to infants < 29 weeks' gestation who achieve the predefined feeding thresholds during their clinical course, and cannot be extrapolated to all extremely preterm infants below 29 weeks' gestation (for example, critically unwell infants with minimal access to human milk). This important population restriction is not clearly emphasised in the trial protocol.

3. The trial enrolls preterm infants with insufficient own mother's milk (OMM), yet OMM intake varies substantially across participants at the point of eligibility. The volume of OMM intake may represent a more strong prognostic determinant of outcomes. The minimisation-based randomisation only balances three pre-specified categorical covariates (neonatal network, gestational-age and SGA), and does not balance participants with respect to volume of OMM intake.

VERSION 1 - AUTHOR RESPONSE

Reviewer
Dr Xiaoping Lei, Affiliated Hospital of Southwest Medical University, Xinhua Hospital, Shanghai Jiao Tong University School of Medicine

2.1 Individual infants may be exposed to two randomised interventions simultaneously, raising the possibility of an interaction between pHDM and human milk fortification status.

Author response: We thank the reviewer for highlighting this point. We agree that, because individual infants may participate in both randomisations, there is the possibility of an interaction between pHDM and human milk fortification status. However, both interventions reflect usual nutritional care that these infants would receive in routine clinical practice, so the trial just randomises which treatment they receive within each comparison. The randomised design allows us to estimate the effects of each intervention while also providing an opportunity to explore whether there is evidence of any interaction (and better than the non-randomised setting where they will receive one from each comparison anyway).

The trial has not been specifically powered to detect an interaction between any of the two interventions, so any interaction analysis will be considered exploratory and interpreted cautiously. Nevertheless, given the size of the trial, we believe this is a valuable opportunity to examine the magnitude and direction of any potential interaction and to identify signals that warrant future investigations.

2.2 The protocol only plans post-hoc subgroup interaction tests with an alpha threshold of 0.20 intended solely for hypothesis-generation. No formal power calculation for detecting interaction effects was pre-specified. Potential synergistic effects between the two interventions may partly distort estimates of main effects, which should have been anticipated a priori.

Author response: The trial was not designed or powered to detect treatment-by-subgroup interactions, as adequately powering such analyses would require a substantially larger sample size and would not have been feasible. The purpose of the

pre-specified subgroup analyses is therefore exploratory rather than confirmatory. The alpha threshold of 0.20 for interaction tests was selected as a screening threshold to identify potential signals of treatment effect heterogeneity that may warrant further investigation in future studies, rather than as a threshold for establishing statistical significance. We have clarified that these analyses are intended solely for hypothesis generation and that any apparent subgroup effects will be interpreted cautiously.

2.3 Multiple interim analyses, two separate randomisation procedures and numerous secondary outcomes increase the risk of type-I error inflation. Only interim efficacy analyses employ the O'Brien-Fleming stopping boundaries; no multiplicity adjustment is mentioned for other secondary or exploratory analyses.

Author response: For each randomisation, type I error for the primary outcome is controlled with respect to the multiple interim analyses using O'Brien–Fleming stopping boundaries. We agree that no multiplicity adjustment is planned across the secondary outcomes and that, consequently, these analyses should not be interpreted as providing confirmatory evidence independently of the primary outcome. We have clarified this in the manuscript and will interpret the secondary outcome analyses as supportive and hypothesis-generating.

We have added the following statement on p. 14: *'No adjustment will be made for multiple testing across secondary outcomes; consequently, these analyses will be regarded as supportive and hypothesis-generating rather than confirmatory.'*

2.4 This study uses event-driven conditional randomisation whereby participants are dynamically screened according to their clinical course. Consequently, trial findings can only be generalised to infants <29 weeks' gestation who achieve the predefined feeding thresholds during their clinical course and cannot be extrapolated to all extremely preterm infants below 29 weeks' gestation (for example, critically unwell infants with minimal access to human milk). This important population restriction is not clearly emphasised in the trial protocol.

Authors' response: We agree that eligibility for each randomisation is determined by the infant meeting the relevant criteria at the appropriate point in their clinical and feeding course, and that the findings should therefore be interpreted in the context of the population eligible for each randomisation. Access to milk is not the issue. The pertinent point is that the trial is aimed at babies who are tolerating enteral feeds, in the case of randomisation 2, a predefined volume.

The trial is designed as a pragmatic study to address two clinically important questions concerning nutritional management in infants born at <29 weeks' gestation. Eligibility for the first randomisation is when there is a shortfall in own mother's milk (OMM). For the second randomisation, infants become eligible when they are receiving 60–120 mL/kg/day of human milk feeds (OMM and/or pHDM), which is standard clinical practice. Therefore, findings will apply to infants who reach these feeding criteria and should not be extrapolated to infants who do not reach this stage or are otherwise ineligible for the randomisation. We feel this is clear in the protocol as its reflected in the study hypotheses and study aims.

2.5 The trial enrols preterm infants with insufficient own mother's milk (OMM), yet OMM intake varies substantially across participants at the point of eligibility. The

volume of OMM intake may represent a more strong prognostic determinant of outcomes. The minimisation-based randomisation only balances three pre-specified categorical covariates (neonatal network, gestational-age and SGA) and does not balance participants with respect to volume of OMM intake.

Authors' response: *We agree that the volume of own mother's milk (OMM) received may be an important prognostic factor. However, randomisation should ensure that OMM volume is not systematically different between comparison groups, particularly given the large sample size. We have additionally used minimisation to balance a limited number of pre-specified, clinically important baseline characteristics (neonatal network, gestational age and small-for-gestational-age status), with adjustment for these variables in the analysis. The purpose of this is to improve the precision of the estimated intervention effect by explaining variation in the outcome. OMM volume at randomisation could be considered a potential minimisation variable but as OMM availability and intake varies over time to do so would not be feasible,*
