# Supplementary material for: Real-world data-enabled, 2-randomisation, adaptive trial to determine the clinical efficacy and safety of widely used enteral feeds in preterm babies: the COLLABORATE trial – protocol
Source: BMJ Open. 2026 Oct 6;16(10):e125163. doi: 10.1136/bmjopen-2026-125163 (PMC13646953; doi:10.1136/bmjopen-2026-125163)
Supplement: Supplementary file 2 [file bmjopen-16-10-s001.docx]

### **SUPPLEMENTARY MATERIALS**

**Section 1. Justification for the study**

**Safety considerations**

**Cow-milk based products:** There is fear among clinicians that highly processed cow-milk based products (PTF and fortifier) may be damaging to the immature intestinal mucosa and increase the risk of NEC. Evidence to support this is however uncertain as we will discuss below in the “Evidence Reviews” section. Feed intolerance and bowel obstruction from "milk curd" impaction have also been described in relation to the use of cow-milk based fortifiers. Conversely, there is fear that without fortification of human milk, nutrient intake will be inadequate, though evidence to support this concern is also lacking ^1^.

### **Pasteurised HDM:** There are worrying safety signals regarding adverse neurodevelopment in relation to use of pHDM. The Canadian Domino Trial randomised very preterm infants to pHDM or Preterm Formula to supplement Own Mothers Milk and found higher neuro-impairment at 18 months in the pHDM arm (27.2% v 16.2%; Adjusted Risk Difference 10.6% [95% CI 1.5%, 19.6]) and a worse mortality/morbidity index (43% v 40%) ^2^.

In a causal inference analysis of five years UK population-based observational data, we found almost the rate of survival without NEC surgery to 34 weeks gestational age was almost 10% lower (Adjusted Risk Difference -9.8% [95% CI -11.4, -8.2]) and a surgical NEC rate that was twice as high in very preterm infants receiving supplemental pHDM, compared with PTF ^3^. These unexpected, counterintuitive findings may reflect unmeasured confounding (i.e., clinician tendency to use pHDM in infants they consider more at risk of NEC) but nonetheless are also justification for a randomised controlled trial.

An observational study from the Canadian Neonatal Network of infants born less than 29 weeks’ gestation, evaluated the relationship between neurodevelopment and enteral feeding ^4^. Infants that received any formula were excluded. These authors found that for each day of reallocating OMM to pHDM was associated with a statistically significant and highly clinically relevant greater odds of cognitive and language impairment at 18-24 months corrected age. This study indicates not only that pHDM should not be considered equivalent to OMM but additionally may be harmful to neurodevelopment.

**Routine fortification:** When added to human milk, current fortifiers provide about 1.1-2.4g additional protein/100 ml in an appropriate ratio with non-protein energy. Preterm milk protein in the first month averages around 1.5g/100 ml but the range is wide (0.7-2.4 g/100 ml). Given that total feed volumes of 180-200 ml/kg are common in the UK, routine fortification risks providing very high protein intakes, exceeding 5g/kg/day, which may be damaging to neurodevelopment ^5^. Concerns have been raised that accelerated weight gain from fortification increases the risks of adverse long-term consequences such as obesity, insulin resistance and hypertension ^1^. The findings of a systematic review and meta-analysis of planned high protein intake after birth for infants born preterm was that this might be harmful for survival, neurodevelopment and metabolism during infancy. The authors concluded that protein intakes ≥3.5 g/kg/d should not be recommended for children born preterm ^5^.

We conducted an analysis of 5 years of whole population data from the UK National Neonatal Research Database (NNRD) in which we compared very preterm infants who had received fortified human milk feeds and those that had not (35,389 infants) (unpublished data). We employed Targeted Maximum Likelihood Estimation, a machine learning approach to address confounding in observational data and maximise causal inferences. We found infants receiving fortified feeds had substantially higher “survival to 34 weeks gestational age without surgical NEC” (Risk Difference 15.1%, 95% CI 14.5 to 15.7). The risk difference increased with decreasing gestational age. “Surgical NEC” was also lower in infants receiving fortified feeds but with a smaller effect size (Risk Difference -1.4%, 95% CI -1.7 to -1.1). These data may reflect residual confounding but also provide justification for a randomised controlled trial (RCT).

### **Evidence reviews**

We searched the Cochrane Central Register of Controlled Trials for reviews and meta-analyses of published studies, and clinical trial registries for ongoing or recently completed trials (ISRCTN Registry; World Health Organisation International Clinical Trials Registry Platform; ClinicalTrials.gov). Collectively, these show there is inadequate evidence of efficacy and safety for the interventions.

**Pasteurised HDM or preterm formula as supplemental feeds:** An initial Cochrane systematic review and meta-analysis comparing pHDM and formula as either sole diet or supplement to OMM was updated to include 12 trials, all carried out in Europe or North America ^6^. The authors included two new trials; the Milk Trial (n=483) conducted in the United States, comparing pHDM with Preterm Formula in extremely preterm infants receiving no or minimal OMM ^7^ and our feasibility study PREMFOOD (NCT01686477) ^8^. The MILK Trial did not complete planned recruitment but showed no significant differences in 2-year neurodevelopment (primary outcome), mortality or bloodstream infection. There was lower medical NEC (secondary outcome) in the pHDM arm. Details of surgical NEC were not provided. In PREMFOOD, we compared fortified human milk feeds (OMM supplemented if required with pHDM), unfortified human milk feeds, and feeds of OMM supplemented if required with PTF. Two infants developed NEC; one with surgical NEC had only received OMM and pHDM; one with medical NEC had only received OMM^8^.

Meta-analysis of data from the 11 RCTs assessing NEC, in which the comparison combined trials of sole and supplementary diets, showed higher risk with formula (Risk Ratio 0.53, 95% confidence interval 0.37 to 0.76; number needed to treat for an additional beneficial outcome 33, 95% CI 20 to 100; 2261 infants). The Cochrane authors conclude that the risk of NEC is halved through use of pHDM in place of formula. However, neither sole nor supplementary comparisons show significant differences in outcomes that would be important corroboration of benefit (mortality; bloodstream infection; neurodevelopment) and no data are presented on surgical NEC. Six of the trials took place over 20 years ago when the patient population differed substantially from that of today; sample sizes were inadequate; methodological quality was poor; and medically managed NEC was included in the outcome, which is an imprecise diagnosis highly liable to ascertainment bias.

The evidence to date therefore, for a reduction in NEC through use of pHDM cannot be regarded as definitive. Robust evidence is required from an adequately powered trial, representative of preterm populations today, and designed to minimise possible ascertainment and other biases.

**Routine protein-carbohydrate fortification of human milk**: The current Cochrane review and meta-analysis compares fortified and unfortified human milk (18 RCT; 1456 infants) and finds no strong statistical evidence of an effect on NEC (13 RCT; 1110 infants; Relative Risk 1.37 [95% CI 0.72, 2.63]) ^9^. Only one RCT in this meta-analysis (245 infants), conducted in 1996, assessed neurodevelopment at 18 months (no difference). Fortification increased in-hospital growth but only two RCT, conducted in 1996 and 1998 respectively, assessed growth at 12-18 months (no differences). The trials were generally small and methodologically weak. Of two other relevant Cochrane reviews, one, comparing trials of human milk fortifier providing different amounts of protein (9 RCT; 861 infants), showed small increases in in-hospital weight gain with higher protein intakes ^10^. The other, comparing early versus late fortification (2 RCT; 237 infants) showed little or no differences in in-hospital growth, surgical NEC, invasive infection, or all‐ cause mortality ^11^. Neither reported long term growth or neurodevelopment.

A review of RCT targeting optimal protein delivery identified two studies evaluating neurodevelopmental outcome ^12^. One randomised <1250g birth weight infants to higher (4.8 g/kg per day) or lower (3.5 g/kg per day) protein intakes for 28 days. Infants receiving the higher intake had better hearing and language scores at 12 and 18 months and greater length gain at 9 months). The other randomised infants <1500 g to 4.2 g/kg per day or 3.6 g/kg per day protein and found no differences in growth or neurodevelopment at 12 and 18 months.

**Section 2. Supplementary tables**

**Supplementary table 1. Study objectives, endpoints and evaluation timepoints**

| **Objectives** | **Endpoints (**Endpoints have been selected taking core neonatal outcome sets into account ^13 14^). | **Evaluation timepoints** |
| --- | --- | --- |
| **Primary** | | |
| To assess. in babies born <29 weeks’ gestation, the efficacy of pHDM compared with Preterm Formula when used as a supplement should there be insufficient milk from their own mother (Own Mother's Milk) on “survival to 34 weeks postmenstrual age without surgical NEC”  To assess, in babies born <29 weeks gestation, if routine cow-milk based protein-carbohydrate fortification of human milk feeds (Own Mother’s Milk and pHDM) compared with no routine fortification, affects “survival to 34 weeks postmenstrual age without surgical NEC” | - Survival without surgical NEC | 34 weeks postmenstrual age |
| **Secondary** | | |
| To assess the efficacy of pHDM compared with Preterm Formula on language and cognitive development at age 2-years, and other outcomes in babies born <29 weeks gestation, when used as a supplement should there be insufficient milk from their own mother (Own Mother's Milk)  To assess if routine cow-milk based protein-carbohydrate fortification of human milk feeds (Own Mother’s Milk and pHDM) in babies born <29 weeks gestation affects language and cognitive development at age 2-years corrected for preterm birth, and other outcomes | - Survival - Surgical NEC - Spontaneous Intestinal Perforation | 34 weeks postmenstrual age |
|  | - Bronchopulmonary dysplasia | 36 weeks postmenstrual age |
|  | - Survival | Age 28 days |
|  | - Survival - Surgery for NEC or NEC related condition after 34 weeks postmenstrual age - Medical NEC - Age in days to achieve enteral intake of 150 ml/kg/day - Treated retinopathy of prematurity - Severe brain injury - Any diagnosis of milk-curd obstruction - Length of neonatal unit stay - Number of episodes of bacterial or fungal bloodstream infection - Number of episodes of bacterial or fungal cerebrospinal fluid infection - Number of episodes of bacterial or fungal urinary tract infection - Number of days of antibiotic treatment - Number of days on parenteral nutrition - Number of days nil by mouth - Weight, length, and head circumference Z-scores - Change from birth in Z-scores for weight, length, and head circumference - Any breast feeding (suckling at breast) - Exclusive breast feeding (suckling at breast) - Receiving any expressed Own Mother’s Milk - Receiving exclusive expressed Own Mother’s Milk - Maximum serum urea, creatine, and alkaline phosphatase - Health resource use | Neonatal unit discharge (or death) |
|  | - Survival without moderate-severe cognitive-language impairment - Survival - Moderate-severe cognitive-language impairment - Cognitive sub-score - Language sub-score - Gross motor function - Hearing impairment - Vison impairment | Age 2-years corrected for preterm birth |
| **In babies with NEC surgery** | - Drain insertion prior to surgery - Diagnosis of short bowel syndrome - Diagnosis of intestinal failure-associated liver disease - Length of bowel resected (cm) - Primary surgical procedure (ileostomy; colostomy; end-to-end anastomosis) | Neonatal unit discharge (or death) |
| **In babies with NEC surgery** | - Number of re-operations (excluding primary operation) | Age 2-years corrected for preterm birth |
| **Tertiary** | | |
| To determine if pHDM and Preterm Formula exert different effects on neurodevelopment through the mechanism of altered cerebral white matter microstructure | - Cerebral white matter microstructure | Term equivalent age (37-44 weeks postmenstrual age) |
| To establish if the additional cost of pHDM to the NHS is justified through a reduction in NEC | - Health resource use | Neonatal unit discharge (or death) |
| To establish if the additional cost of fortifier, generates savings through improved survival to age 2-years corrected for preterm birth without moderate-severe cognitive-language impairment | - Health resource use (subject to future funding) | Age 2-years corrected for preterm birth |
| To collect and store faecal samples from participants recruited in Edinburgh who are enrolled in randomisation 1, for future mechanistic studies | - N/A subject to future funding | N/A |

**Supplementary table 2****. Schedule of assessments**

| **Timepoint →**  **Assessment ↓** | **On admission to neonatal care** | **28 days postnatal age** | **34 weeks postmenstrual age** | **36 weeks postmenstrual age** | **Discharge** | **37-44 weeks postmenstrual age** | **2 years corrected for prematurity**  ***(23.5–27.5 months)*** |
| --- | --- | --- | --- | --- | --- | --- | --- |
| **Eligibility** | X |  |  |  |  |  |  |
| **Consent** | X |  |  |  |  |  |  |
| **Randomisation*** | X |  |  |  |  |  |  |
| **Highest recorded serum urea, creatinine, alkaline phosphatase** |  |  |  |  | X |  |  |
| **Clinical endpoints from NNRD data** |  | X | X | X | X |  |  |
| **Additional clinician recorded endpoints** |  |  |  |  | X |  |  |
| **MRI**** |  |  |  |  |  | X |  |
| **Stool sample**** | X | X | X |  |  | X |  |
| **PARCA-R***** |  |  |  |  |  |  | X |
| **Adverse Reactions** |  | X | X |  | X | X |  |

** When the participant meets the relevant inclusion/exclusion criteria for each randomisation*

*** Only participants in the mechanistic sub-study*

**** To be completed using the digital PARCA-R where possible (facilitated through Imperial Clinical Trials Unit)*

**Supplementary table 3. Interim efficacy analyses**

| **Proportion recruited** | **50%** | | | **75%** | | **100%** | |
| --- | --- | --- | --- | --- | --- | --- | --- |
| Randomisation 1, n | 954 | | | 1431 | | 1908 | |
| Randomisation 2, n | 1063 | | | 1594 | | 2126 | |
| Critical value thresholds (Z-scale) | | 2.863 | 2.337 | | 2.024 | |  |

### **Section 3. Mechanistic Study**

COLLABORATE includes an embedded mechanistic, single-centre, sub-study that will use magnetic resonance imaging (MRI) to determine whether pHDM and Preterm Formula have different effects on cerebral white matter maturation, the hallmark feature of encephalopathy of prematurity, and hence impact the development of brain networks that underpin cognition. We will explore the potential differential effects of the interventions on cortical morphology and microstructure. This sub-study will involve 120 infants participating in COLLABORATE at the Royal Infirmary of Edinburgh.

The gut microbiota is thought to play a role in human health and disease, including child development ^15^; it is modified by age at birth, sex, mode of delivery, antibiotic exposure, and feed type. The microbiome may mediate interactions of the gut-brain axis, including in preterm infants ^16-18^. For infants enrolled in the mechanistic study in Edinburgh, faecal samples will be collected at enrolment and weekly to 34 weeks. Samples will be processed and stored at -80°C for later analyses subject to ethics approval and funding, exploring mechanisms linking nutritional exposures to brain development through gut-brain interactions.

Based on computational modelling and prior precedent from neonatal neuroprotection trials, a study of 60 infants in each treatment arm will enable detection of a 10% difference in fractional anisotropy with 80% power, 2-sided 5% significance ^19^. Diffusion tensor imaging (DTI) data will be analysed using Functional MRI of the Brain Software Library ^20^ and DTI-ToolKit. Data will be corrected for phase encoding distortions, eddy-induced distortions and motion using the topup-eddy algorithm, using T2 structural volumes rigidly registered to b0 maps and assuming a bandwidth of zero (no phase-encoding) ^21^. A diffusion tensor model will be fitted to each voxel and the eigenvalues and eigenvectors used to convert the corrected diffusion weighted images into diffusion tensor volumes. Tensor-based image registration will be used to produce a population-specific diffusion tensor template. From this template the mean Fractional Anisotropy (FA) volume is derived and thinned by perpendicular non-maximum suppression to create the mean white matter tract skeleton, thresholder at FA > 0·15 to exclude peripheral tracts. All participants’ diffusion tensor volumes will be registered to the diffusion tensor template and FA and other parameters extracted and projected onto the white matter tract skeleton.

### **Section 4. Statistical considerations**

### **Internal pilot:** We will run an internal pilot to assess recruitment rate; co- enrolment rate; and withdrawal of consent rate (Supplementary Table 4). The internal pilot will run for 9 months (6-15 months).

We need adequate recruitment for each of the comparisons to be powered to detect the MCID of 0.05. We estimate recruitment will increase over the first 9 months from 25 per month to an average of 97 by month 6. The proportions of babies taking part in both randomisations is important to monitor as this will impact the power of the study. If less than 30% take part in both randomisations, then power will be <80%. We will also monitor the sample size assumption for missing data which will occur when carers/parents withdraw consent for use of their baby’s information in NNRD to be analysed. The internal pilot will run seamlessly into the main study if all three criteria below are met. The Trial Steering Committee will be responsible for assessing the internal pilot outcomes and provide guidance to the trial team.

**Exploratory analyses:** Exploratory analyses will be undertaken to estimate how the volume of supplementary feed (pHDM or formula) and the time of introduction impacts outcome. This will be performed using Parametric G-formula, a method to estimate the effects of treatment strategies adjusting for time-varying confounders affected by prior exposures.

### **Safety analyses:** We will tabulate adverse events by intervention arms for each randomisation. For safety events of special interest (death, surgical NEC, medical NEC, infection) we will follow the principle of the primary analysis. For all other events we will calculate the incidence rate ratio and present these graphically using dot plots.

Binary secondary and key safety outcomes (death, surgical NEC, medical NEC, infection) will be analysed using the same principled approach as the primary outcome. The age 2-year PARCA-R will also be evaluated as a continuous outcome and the between arm mean difference estimated using the GEE model with normal distribution and identity link function adjusting for minimisation variables.

# **Secti****on 5. Cost effectiveness study analyses**

### As COLLABORATE will be viewed as providing definitive evidence by the medical community, there is a pressing need to establish the value for money of pHDM versus preterm formula, especially due to the large cost difference between the two interventions and the high costs of NEC treatment ^22^. NHS secondary care utilisation from randomisation until neonatal unit discharge will be obtained from the NNRD, as used in other successful economic evaluations ^23 24^.

To calculate the costs associated with daily neonatal care received by each baby, we will multiply the number of days spent receiving each level of care, by level-specific national average bed day costs sourced from National Schedule of NHS Costs ^25^. Such costs are estimated by assigning each level of care a healthcare resource group (HRG) code. HRG are groupings of clinically meaningful activities made primarily on the basis of diagnosis and procedure codes, and within the NHS, are the ‘units’ of healthcare for which providers receive payment. Costs assigned to each HRG are based on nationally estimated reference costs developed into adequately cover the cost of providing high-quality and cost-effective care. For this analysis, the neonatal bed day costs use will be intensive care (HRG XA01Z), high dependency care (HRG XA 02Z), special care without carer resident alongside baby (HRG XA03Z), special care with carer resident alongside baby (HRG XA04Z) and normal care (HRG XA05Z).

Given that some major clinical activities are not included in these neonatal care HRG we will identify high-cost non-routine procedures captured within the NNRD, considered to be important in this population. For example, types of neonatal surgery will be identified from their corresponding Operating Procedure Code Standard and International Statistical Classification of Diseases and Health Related Problems (ICD-10) codes for costing purposes and unit costs obtained from the NHS National Schedule of Costs.

For both trial comparisons, cost results will be reported as means with standard deviations. Mean differences, together with 95% confidence intervals, will be estimated using multilevel generalised gamma linear models with a random intercept at the neonatal unit level. For comparison 2 (fortification versus no fortification), results will additionally be presented by subgroup: no clinical need for supplementation of mother’s own milk; and if supplementation is required, by randomisation group in comparison 1, i.e. pHDM and Preterm Formula. Costs will be presented alongside the key primary and secondary outcomes by intervention arm in both comparisons with associated uncertainty. We will use the NNRD to determine the number of extremely preterm babies and hence estimate the NHS budget implications for the UK of all study interventions. This approach will enable various stakeholders (e.g. parents, clinicians and commissioners) to contemplate the impact of pHDM and milk fortification on the outcomes of most relevance to them.

If trial interventions result in better 2-year cognitive-language outcomes, we will develop a decision analytical model to estimate the cost-effectiveness of the intervention up to 18 years of age. This analysis will be conducted from an NHS and societal perspective and the main outcome measure in the economic evaluation will be the child’s quality-adjusted life years. A Markov model will be constructed representing the natural history of infants to extrapolate the within-trial cost-effectiveness results using annual cycles. The structure of the model will be established and agreed within the research team. Observed outcomes and health care resource utilisation for randomised infants will be used to inform the characteristics of a hypothetical cohort entering the model. Transition probabilities indicating movement across health states during the first two years will be obtained from the trial, whereas transition probabilities after the second year will be informed through literature searches. Health care costs and health-related quality of life estimates incurred annually in each health state after neonatal care discharge will be obtained from the literature. Data on informal care and impact on productivity of parents to inform cost parameters in the model will also be obtained from the literature if available.

To be able to follow-up trial participants using routine sources, we will seek parent consent to access long-term data. This will enable future data linkage to the Department for Education National Pupil Database on performance in Key Stage 1 and Special Educational Needs, and use of health services and mortality through Hospital Episode Statistics.

**REFERENCES**

1. Modi N. The implications of routine milk fortification for the short and long-term health of preterm babies. *Semin Fetal Neonatal Med* 2021;26(3):101216. doi: 10.1016/j.siny.2021.101216 [published Online First: 20210225]

2. O'Connor DL, Gibbins S, Kiss A, et al. Effect of Supplemental Donor Human Milk Compared With Preterm Formula on Neurodevelopment of Very Low-Birth-Weight Infants at 18 Months: A Randomized Clinical Trial. *JAMA* 2016;316(18):1897–905. doi: 10.1001/jama.2016.16144

3. Chehrazi M, Lanoue J, Ougham K, et al. Outcomes in very preterm infants receiving an exclusive human milk diet, or their own mother's milk supplemented with preterm formula. *Early Hum Dev* 2023;187:105880. doi: 10.1016/j.earlhumdev.2023.105880 [published Online First: 20231021]

4. Bando N, Yoon EW, Beltempo M, et al. Association of Enteral Feed Type with Neurodevelopmental and Neonatal Outcomes among Infants Born Preterm. *J Pediatr* 2025;281:114536. doi: 10.1016/j.jpeds.2025.114536 [published Online First: 20250313]

5. Das S, McClintock T, Cormack BE, et al. High protein intake on later outcomes in preterm children: a systematic review and meta-analysis. *Pediatr Res* 2025;97(1):67–80. doi: 10.1038/s41390-024-03296-z [published Online First: 20240610]

6. Quigley M, Embleton ND, Meader N, et al. Donor human milk for preventing necrotising enterocolitis in very preterm or very low-birthweight infants. *Cochrane Database Syst Rev* 2024;9(9):CD002971. doi: 10.1002/14651858.CD002971.pub6 [published Online First: 20240906]

7. Colaizy TT, Poindexter BB, McDonald SA, et al. Neurodevelopmental Outcomes of Extremely Preterm Infants Fed Donor Milk or Preterm Infant Formula: A Randomized Clinical Trial. *JAMA* 2024;331(7):582–91. doi: 10.1001/jama.2023.27693

8. Mills L, Chappell KE, Emsley R, et al. Preterm Formula, Fortified or Unfortified Human Milk for Very Preterm Infants, the PREMFOOD Study: A Parallel Randomised Feasibility Trial. *Neonatology* 2024;121(2):222–32. doi: 10.1159/000535498 [published Online First: 20231213]

9. Brown JV, Lin L, Embleton ND, et al. Multi-nutrient fortification of human milk for preterm infants. *Cochrane Database Syst Rev* 2020;6(6):CD000343. doi: 10.1002/14651858.CD000343.pub4 [published Online First: 20200603]

10. Gao C, Miller J, Collins CT, et al. Comparison of different protein concentrations of human milk fortifier for promoting growth and neurological development in preterm infants. *Cochrane Database Syst Rev* 2020;11(11):CD007090. doi: 10.1002/14651858.CD007090.pub2 [published Online First: 20201120]

11. Thanigainathan S, Abiramalatha T. Early fortification of human milk versus late fortification to promote growth in preterm infants. *Cochrane Database Syst Rev* 2020;7(7):CD013392. doi: 10.1002/14651858.CD013392.pub2 [published Online First: 20200729]

12. Brown LD, Stremming J, Robinson DT. Targeting optimal protein delivery in parenteral and enteral nutrition for preterm infants: a review of randomized, controlled trials. *J Perinatol* 2024;44(5):603–11. doi: 10.1038/s41372-023-01847-6 [published Online First: 20231220]

13. Klerk DH, van Varsseveld OC, Offringa M, et al. Core Outcome Set for Necrotizing Enterocolitis Treatment Trials. *Pediatrics* 2024;153(6) doi: 10.1542/peds.2023-065619

14. Webbe JWH, Duffy JMN, Afonso E, et al. Core outcomes in neonatology: development of a core outcome set for neonatal research. *Arch Dis Child Fetal Neonatal Ed* 2020;105(4):425–31. doi: 10.1136/archdischild-2019-317501 [published Online First: 20191115]

15. Vaher K, Bogaert D, Richardson H, et al. Microbiome-gut-brain axis in brain development, cognition and behavior during infancy and early childhood. *Developmental Review* 2022;66

16. Neu J, Stewart CJ. Neonatal microbiome in the multiomics era: development and its impact on long-term health. *Pediatr Res* 2025;98(4):1223–31. doi: 10.1038/s41390-025-03953-x [published Online First: 20250228]

17. Cryan JF, O'Riordan KJ, Cowan CSM, et al. The Microbiota-Gut-Brain Axis. *Physiol Rev* 2019;99(4):1877–2013. doi: 10.1152/physrev.00018.2018

18. Vaher K, Cabez MB, Parga PL, et al. The neonatal gut microbiota: A role in the encephalopathy of prematurity. *Cell Rep Med* 2024;5(12):101845. doi: 10.1016/j.xcrm.2024.101845 [published Online First: 20241204]

19. Ball G, Boardman JP, Arichi T, et al. Testing the sensitivity of Tract-Based Spatial Statistics to simulated treatment effects in preterm neonates. *PLoS One* 2013;8(7):e67706. doi: 10.1371/journal.pone.0067706 [published Online First: 20130703]

20. Jenkinson M, Beckmann CF, Behrens TE, et al. Fsl. *Neuroimage* 2012;62(2):782–90. doi: 10.1016/j.neuroimage.2011.09.015 [published Online First: 20110916]

21. Winkler AM, Ridgway GR, Webster MA, et al. Permutation inference for the general linear model. *Neuroimage* 2014;92(100):381–97. doi: 10.1016/j.neuroimage.2014.01.060 [published Online First: 20140211]

22. Pokhrel S, Quigley MA, Fox-Rushby J, et al. Potential economic impacts from improving breastfeeding rates in the UK. *Arch Dis Child* 2015;100(4):334–40. doi: 10.1136/archdischild-2014-306701 [published Online First: 20141204]

23. Pillay T, Modi N, Rivero-Arias O, et al. Optimising neonatal service provision for preterm babies born between 27 and 31 weeks gestation in England (OPTI-PREM), using national data, qualitative research and economic analysis: a study protocol. *BMJ Open* 2019;9(8):e029421. doi: 10.1136/bmjopen-2019-029421 [published Online First: 20190822]

24. Yang M, Campbell H, Pillay T, et al. Neonatal health care costs of very preterm babies in England: a retrospective analysis of a national birth cohort. *BMJ Paediatr Open* 2023;7(1) doi: 10.1136/bmjpo-2022-001818

25. National Cost Collection for the NHS: NHS England; [Available from: <https://www.england.nhs.uk/costing-in-the-nhs/national-cost-collection/>.
